# Use of chicken feather meal fermented with *Bacillus subtilis* in diets to increase the digestive enzymes activity and nutrient digestibility of silver pompano *Trachinotus blochii* (Lacepede, 1801)

**DOI:** 10.12688/f1000research.26834.2

**Published:** 2021-03-25

**Authors:** Adelina Adelina, Feli Feliatra, Yusni Ikhwan Siregar, Iskandar Putra, Indra Suharman

**Affiliations:** 1 Department of Aquaculture, Faculty of Fisheries and Marine Science, Universitas Riau, Pekanbaru, 28293, Indonesia; 2 Department of Marine Science, Faculty of Fisheries and Marine Science, Universitas Riau, Pekanbaru, 28293, Indonesia

**Keywords:** Bacillus subtilis, digestibility, diet, feather meal, Trachinotus blochii

## Abstract

**Background**: Feather has the potential to be used as a fish feed ingredient because it has high protein content (80-85%), and is rich in amino acids arginine, leucine, isoleucine and valine. However, the protein consists mainly of keratin, which is classified as fiber that is difficult to digest. Therefore, to improve digestibility, the keratin protein is degraded using microbial *Bacillus subtilis*. This study aimed to determine the digestibility of fermented feather meal (FFM) in silver pompano ( *Trachinotus blochii*) diets and to observe the histological structure of their intestines after digestion.

**Methods**: The method used was a one factor experiment with five treatments and three replications each, which were: diet without FFM, diet containing 10% FFM, 20%, 30% and 40%. The diets were given to juvenile silver pompano (with average body weight of 8.56 ± 0.18 g) and stocked in 15 similar 20-L plastic jars with 10 fish per jar in a density of 100 L capacity container. The experimental diets were given three times daily at approximately 8.00 AM, 12.00 PM and 5.00 PM to apparent satiation for 60 days.

**Results**: The results showed that the use of FFM increased the activity of digestive enzymes (protease and lipase), but reduced the amylase activity of silver pompano, which was significantly different between treatments (P <0.05). Meanwhile, the diet containing 20% FFM produced the highest feed and protein, which are 37.05% and 67.24%, respectively. This was significantly different from other treatments (P <0.05), and was effectively absorbed by fish intestines.

**Conclusion:** The addition of chicken feather meal fermented with *Bacillus subtilis* could increase the activity of protease and lipase enzymes and nutrient digestibility of silver pompano but not amylase activity.

## Introduction

Feathers are by-products from chicken slaughterhouses, and production has continued to increase along with the rise in population and increased demand for chicken as a source of animal protein. Feather waste with poor management causes problems for human health because feathers are a source of odors and disease. Feathers are very difficult to degrade, and the decomposition process takes a long time, which has an impact on soil quality ^1^.

Utilization of feather waste is not optimal, and thus far, only a small portion is used to make dusters, vehicle seats, plant fertilizer, handicrafts, and shuttlecocks. In terms of nutrient content, feather meals are high in protein (80–85%) and rich in amino acids arginine, cystine, leucine, isoleucine, and valine ^2^. Hence, they have the potential to be used as fish feed ingredients. However, 90% of the protein is composed of beta-keratin, a component that is classified as a difficult to digest fiber ^3^. Keratin consists of cystine disulfide and hydrogen bonds, as well as hydrophobic interactions. The disulfide bonds between the cystine amino acids make the protein difficult to digest by proteolytic enzymes ^4^. Therefore, to utilize feather as fish feed ingredient, the keratin needs to be degraded first.

Heterotrophic bacteria found in the ocean have the potential to break down complex organic matter into simpler components ^5^. In addition, a hydrolysis technique using microbes has been found to degrade keratin in feather protein using *Bacillus licheniformis* ^2^ *and Bacillus subtilis* ^6^. The keratinase enzyme produced by *B. licheniformis* can also hydrolyze various proteins including collagen, elastin, and keratin, as well as increase the digestibility of feather meal protein ^2^. High or low digestibility of feather meal in feed is largely determined by the role of digestive enzymes. Bacteria of the genus *Bacillus* have a major role in the digestive tract of fish ^7^ because they produce several extracellular enzymes such as proteases, lipases, and amylases. These enzymes catalyze the breakdown of complex nutrients (proteins, lipids and carbohydrates) into simple components, thereby increasing digestibility ^6^. Adelina *et al*. ^8^ showed that the digestibility of feed protein containing feather meal in white snapper ( *Lates calcarifer*, Bloch) was 39.09%, increasing to 48.75% after fermentation using *Bacillus subtilis*.

Measuring the performance of feed absorption in fish digestive systems involves observing the histological structure of the intestine, which is an important organ responsible for digestion and absorption ^9^. In addition, feed of good quality is not only based on the nutritional constituents, but also on other components that aid easy digestion and absorption. As long as the food is in the intestine, the nutrients are broken down by various enzymes into a form that can be absorbed, and then enter the circulatory system for use in physiological processes and growth.

Therefore, this study aimed to examine the digestibility and absorption of feed containing fermented feather meal (FFM) in the intestine of silver pompano ( *Trachinotus blochii*, Lacepede). This fish was chosen because of its important economic value; its market demand is quite high at local and international levels, particularly in Singapore, Japan, Taiwan, Hong Kong, and China ^10^.

## Methods

### Ethical statement

Ethical approval for the study was obtained from Syiah Kuala University Research and Ethics Guidelines, Section of Animal Care and Use in Research (Ethic Code No: 958 /2015). All efforts were made to lessen harm to the animals by applying the Syiah Kuala University Research and Ethics Guidelines, Section of Animal Care and Use in Research ethical code.

### Feather meal fermentation using *Bacillus subtilis*

Broiler chicken feathers were collected from the local market, washed with clean water, sterilized by steaming at 100°C for 15 minutes, cooled at room temperature, and then dried in an oven at 60°C for six hours. Dried chicken feathers were grinded using a disk mill into feather meal. The material used for fermentation was *Bacillus subtilis* (GenBank accession no. JX188065.1), which was collected in the Marine Microbiology Laboratory of the Faculty of Fisheries and Marine Science, University of Riau, Indonesia ^11^. Isolated *B. subtilis* was vortexed for inoculum, then transferred by dropping as much as 50 μ into a nutrient agar (NA) medium. The bacteria were flattened using a sprier, then incubated for 24 hours. The bacteria were then purified four times on the NA medium to obtain pure *B. subtilis* colonies. Pure *B. subtilis* was transferred to nutrient broth liquid media for propagation. *B. subtilis* was then used as a fermenter for chicken feather fermentation. The fermentation process was carried out by sterilizing the feathers in an oven at 100°C for 15 minutes, then cooling. The sterile outcome was placed in five Petri dishes (2 g each), adding 10 ml of pure *B*. *subtilis*, and incubating at 50°C, pH 8 for 72 hours ^12^. The FFM was then ready to be used as fish feed ingredient.

### Diet preparation

The experimental diet used was artificial feed with a protein content of about 40–41%. The ingredients were purchased from the local market and weighed according to the formulations in Table 1, mixed until homogeneous, molded into pellets, and then dried in an oven at 60°C. The dry pellets were then analyzed in accordance AOAC methods ^13^. Crude protein (Nx6.25) was measured using the micro-Kjeldahl nitrogen determination method. Crude lipid was determined by the ether-extraction method. Moisture was determined by oven-drying at 105°C until a constant weight was achieved. Ash content was measured after placing the samples in a muffle furnace at 550°C for 24 hours. The results of the proximate composition are shown in Table 1. 

**Table 1.** Formulation and proximate composition of experimental diets.

Ingredient (%)	Diet P0 (0% FFM)	Diet P1 (10% FFM)	Diet P2 (20% FFM)	Diet P3 (30% FFM)	Diet P4 (40% FFM)
Fish meal FFM ^1^ Tofu waste Wheat meal Vitamin mix ^2^ Mineral mix ^3^ Fish oil Chromic oxide	65 0 23 6 2 2 2 1	48 10 29 7 2 2 2 1	32 20 34 8 2 2 2 1	16 30 38 10 2 2 2 1	0 40 41 13 2 2 2 1
Proximate composition (%)					
Crude protein Crude lipids Moisture Ash Crude fiber NFE ^4^ Chromic oxide	41.45 2.08 7.49 9.18 7.51 32.29 0.69	41.88 2.27 7.04 12.63 7.66 29.52 0.80	41.46 2.21 6.62 9.17 7.88 32.66 0.78	40.93 2.33 9.29 5.84 7.34 34.27 0.80	40.34 2.46 7.16 5.86 7.78 36.40 0.74

^1^ FFM = fermented feather meal. ^2^ Vitamin mix (mg/100 g diet): thiamin 5.0; riboflavin 5.0; Ca-pantothenate 10.0; niacin 2.0; pyridoxin 4.0; biotin 0.6; folic acid 1.5; cyanocobalamin 0.01; inositol 200; ρ-aminobenzoic acid 5.0; menadion 4.0; vit A palmitate 15.0; chole-calciferol 1.9; α-tocopherol 20.0; cholin chloride 900.0. ^3^ Mineral mix (mg/100 g diet): KH2PO4 412; CaCO3 282; Ca (H2PO4) 618; FeCl3.4H2O 166; ZnSO4 9.99; MnSO4 6.3; CuSO4 2; CuSO4.7H2O) 0.05; KJ 0.15; Dextrin 450; Cellulose 553.51. ^4^ NFE = nitrogen-free extract; calculated = 100-(%CP+%CL+%moisture+%ash+%CF).

### Study design

This experiment used a completely randomized design (CRD). Five levels of FFM were tested, namely: P0 (0% FFM), P1 (10% FFM), P2 (20% FFM), P3 (30% FFM), and P4 (40% FFM). Every treatment was replicated three times.

### Feeding trial and digestibility study

The juvenile silver pompano were purchased from Batam Marine Aquaculture Center (BPBL) located in Setoko islands, Batam city were placed and acclimatised in 400- L plastic tanks for a week. After the acclimation, juvenile silver pompano with mean body weight of 8.56 ± 0.18 g were randomly distributed into 15 similar 20-L plastic jars filled with 15-L water (10 fish per jar with three replication per treatment). They were then fed three times daily at 8.00 AM, 12.00 PM, and 17.00 PM until apparent satiation. All leftover feeds were collected after one hour, and subsequently, fresh feces were collected by siphoning after 4–5 hours. Feces were also collected every morning before feeding time. All fecal samples were pooled until sufficient amounts had been obtained for chemical analysis. They were then completely dried in an oven at 60°C, ground using a laboratory grinder, and kept in a refrigerator at 16°C until further analysis. The protein, lipid, carbohydrate, and chromic oxide content of the feed and feces were analyzed using AOAC methods ^13^ as mentioned above. After 60 days of the feeding test, two fish were taken from each plastic tank, anesthetized with tricaine methanesulfonate (MS-222) and their intestines were removed and frozen at -80°C until analysis for the activity of protease, lipase, and amylase enzymes. Intestinal retrieval was carried out 18 hours after consumption of the last feed ^14^.

### Variables observed and measured

a. Protease, lipase, and amylase enzymes activity of fish

Two fish were taken from each test or six fish from each treatment, and their intestines were removed. The intestine was weighed, and a Tris buffer solution was added (20 mM Tris HCl, 1 mM EDTA, 10 mM CaCl2, pH 7.5) at a ratio of 10%. It was then put into an effendorf tube and centrifuged at 12,000 rpm for 10 mins, at a temperature of 4°C. The supernatant was taken and the activities of protease, lipase, and amylase enzymes were analyzed. Protease activity was determined using the method that was previously used by Bergmeyer *et al*. ^15^. 0.01 M borate buffer (pH 8) was added into 2% casein substrates. The mixture was incubated at 37°C for 10 min, mixed with 0.1 M TCA and re-incubated at 37°C for 10 min. The mixture was centrifuged at 4000 rpm for 10 min, mixed with 5.0 mL Na _2_CO _3_ and 1 mL reagent Follin (1:2). The absorbance was checked using spectrophotometer (UV-1800, Shimadzu Europa, Duisburg, Germany) at 578 nm. Lipase activity was analyzed according to Borlongan ^16^. 1.5 mL olive oil was mixed with 1.5 mL of 0.1 M HCl tri buffer at pH 8.0. The mixture was incubated for 6 h at 37°C, pursued by adding 3 mL ethyl alcohol (95%). 0.01 N NaOH was applied to titrate the mixture by using 0.9% thymolphthalein ethanol as an indicator. Analysis of amylase activity was based on Bernfield ^17^. The reaction mixture consisted of citra buffer solution (pH 5.7) and starch solution 1%. The mixture was incubated for 30 min at 20°C, mixed with 2 mL DNS and poached for 5 min. The absorbance was measured by using a spectrophotometer (UV-1800, Shimadzu Europa, Duisburg, Germany) at 550 nm.

b. Fish intestine histology

Histological analysis was conducted to observe the structure of the intestinal wall after being given a test feed containing FFM. At the end of maintenance, the intestines of the test fishes from each treatment were removed, and histologically prepared to observe the condition using the Olympus binocular microscope model CX 21. The observations were then compared to determine the differences between the treatments. Goblet cells, congestion and hemorrhage in the intestinal tissues among treatments was compared. The preparation method was carried out according to Dellman *et al.* ^18^ The fish was dissected and their intestines were taken cut to a thickness of 0.5 cm and then fixed in 10% formalin solution for 24 hours. The dehydration process was carried out, which begins by putting the sample in a bottle containing 30%, 50%, 70%, 90% and 100% alcohol for 45 minutes each, which aims to remove the moisture content from the cells / tissues and replace it with alcohol. Then the sample was inserted into xylol 1 and xylol 2 for 45 minutes each for dealcoholization.

### Data analysis

Enzyme activity data (protease, lipase, and amylase) and feed digestibility were analyzed using analysis of variance (ANOVA). Duncan’s Multiple Range Test (DMRT) was used to determine the differences between the treatments. The analysis was performed using SPSS v. 18.0 software. Alphabetical notations (a, b, c) are used to mark significant differences at a significance level of p<0.05. Histology of the intestine was descriptively analyzed by comparing the conditions for each treatment.

## Results

### Protease, lipase, and amylase enzyme activity

**Figure 1.**
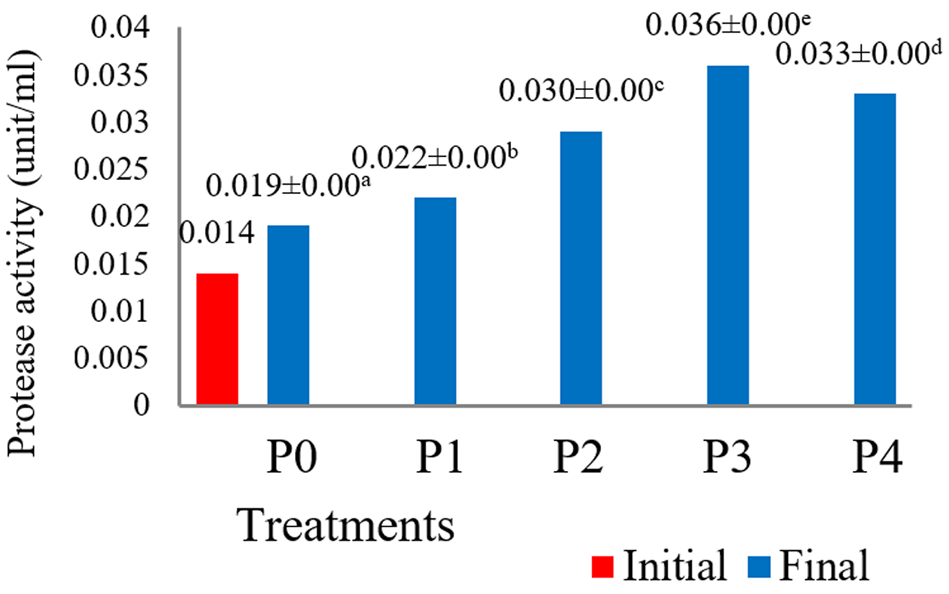
Changes in protease enzyme activity in the intestine of silver pompano. P0 = FFM 0%, P1 = FFM 10%, P2 = FFM 20%, P3 = FFM 30%, and P4 = FFM 40%.

**Figure 2.**
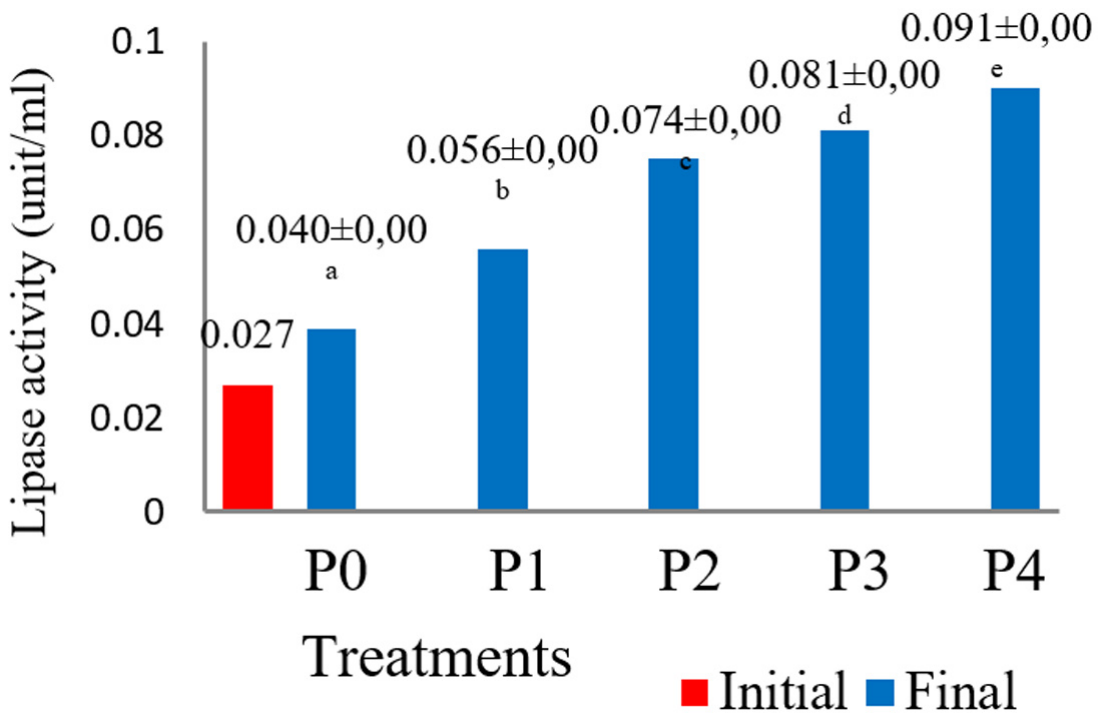
Changes in lipase enzyme activity in the intestine of silver pompano. P0 = FFM 0%, P1 = FFM 10%, P2 = FFM 20%, P3 = FFM 30%, and P4 = FFM 40%.

**Figure 3.**
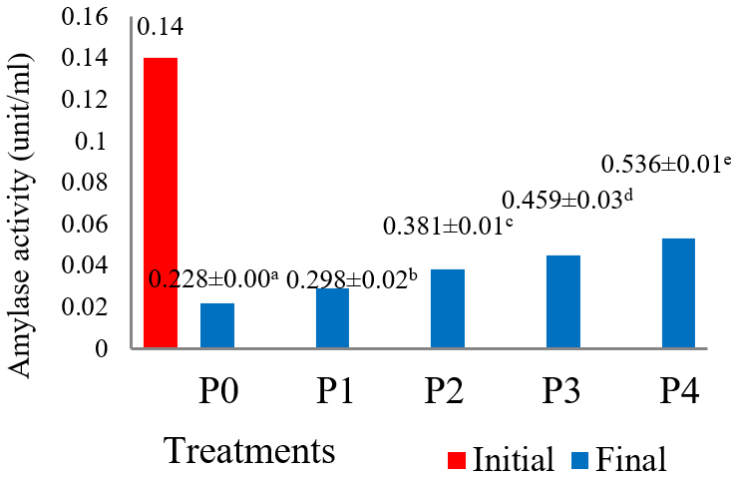
Changes in amylase enzyme activity in the intestinal of silver pompano. P0 = FFM 0%, P1 = FFM 10%, P2 = FFM 20%, P3 = FFM 30%, and P4 = FFM 40%.

Protease, lipase, and amylase enzymes activity measurements in the intestine of silver pompano with FFM feed can be seen in Figure 1, Figure 2, and Figure 3, respectively. The results showed that protease and lipase enzyme activities were higher at the end of the observation compared to the beginning. In contrast, amylase activity was higher at the beginning than the end.

Protease, lipase, and amylase enzyme activities in the intestine of fish fed with FFM (P1, P2, P3 and P4) were found to be higher than in those without FFM (P0) and were significantly different (p<0.05) between the treatments. The highest protease activity was found in the P3 (30% FFM) diet, which was significantly different from other treatments (p<0.05). The highest lipase and amylase enzyme activities were found in the P4 (40% FFM diet), which were significantly different from other treatments (p<0.05).

### Digestibility of protein, lipid, and carbohydrate

Feed digestibility is strongly influenced by digestive enzymes that breakdown nutrients and make them easily absorbed. The feed containing FFM resulted in the levels of digestibility of proteins, lipids and carbohydrates shown in Table 2.

**Table 2.** Feed and nutrient (protein, lipid and carbohydrate) digestibility.

Parameter	Diet P0 (FFM 0)	Diet P1 (FFM 10)	Diet P2 (FFM 20)	Diet P3 (FFM 30)	Diet P4 (FFM 40)
Protein digestibility (%)	51.45±2.97 ^a*^	61.78±1.77 ^b^	68.25±2.12 ^c^	62.97±2.61 ^b^	59.48±2.42 ^b^
Lipid digestibility (%)	42.29±1.91 ^a*^	43.19±0.32 ^a^	45.21±2.13 ^a^	42.19±1.73 ^a^	41.76±1.76 ^a^
Carbohydrate digestibility (%)	45.01±2.64 ^a*^	53.16±1.44 ^b^	55.19±1.64 ^b^	54.05±2.75 ^b^	52.85±2.72 ^b^
Feed digestibility (%)	22.07±1.93 ^a*^	31.48±2.93 ^b^	37.38±1.72 ^c^	33.82±1.91 ^bc^	29.05±2.28 ^b^

*Data values are the mean and standard deviation. The means with different superscripts (a, b, c) in the same row were significantly different (p < 0.05).

The fermentation of feather meal using *B*. *subtilis* resulted in increased digestibility. The feed without FFM (P0) had the lowest digestibility compared to those that contain FFM (P1, P2, P3, and P4) and was significantly different from the other treatments (p<0.05). Feed with 20% FFM (P2) had the highest digestibility but was not significantly different (p>0.05) from feed containing 30% FFM (P3).

The use of different FFM percentages in feed resulted in different levels of digestibility of nutrients (proteins, lipids, and carbohydrates). The feed without FFM (P0) had the lowest protein digestibility compared to feed containing FFM (P1, P2, P3, and P4) and was significantly different from the other treatments (p<0.05). Meanwhile, the feed containing 20% FFM (P2) had the highest digestibility, and was significantly different (p<0.05) from the other treatments. 30% FFM (P3) and 40% FFM (P4) feeds had lower protein digestibility compared to those containing 20% FFM (P2). Feed without FFM (P0) had lipid digestibility that was not significantly different (p>0.05) from those with FFM (P1, P2, P3 and P4). In addition, feed without FFM (P0) had the lowest carbohydrate digestibility compared to feed with FFM (P1, P2, P3, and P4) and was significantly different from the other treatments (p<0.05), whereas feed containing FFM (P1, P2, P3 and P4) had carbohydrate digestibility that was not significantly different between treatments (p>0.05).

### Intestine histology of silver pompano

**Figure 4.**
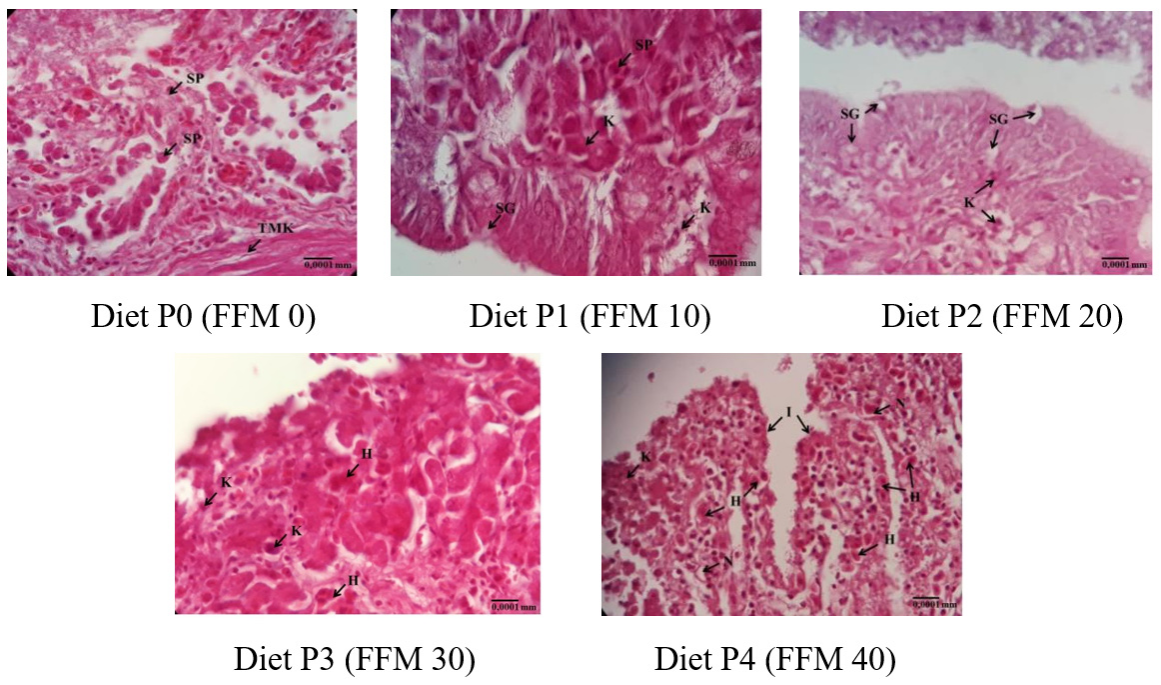
The intestinal histology of silver pompano fed with fermented feather meal. TMK = tunica muscularis, SP = leftover feed, SG = goblet cells (mucous cells), K = congestion, H = hemorrhage, I = irritation/erosion, N = necrosis coloration, HE = Hematoxylin Eosin, enlargement 1000x.

The intestinal histology of silver pompano fed with FFM is presented in Figure 4.

Figure 4 shows that fish fed without FFM (P0) have normal intestinal conditions and can easily digest food. The intestine of P1 treated fish (10% FFM) was seen to produce goblet cells that facilitate absorption. Those fed with 20% FFM (P2) produced more goblet cells in the intestine, suggesting they are able to effectively absorb feed. The use of 30% FFM in feed (P3) resulted in congestion (capillary tissue full of blood) and hemorrhage (blood spreads to the tissues) in the intestinal tissue of the fish. This is characterized by erythrocytes that leak out of blood vessels into the mucus membrane of intestinal tissue. Furthermore, the use of more FFM in feed (40%, P4) caused damage to the intestinal tissue (necrosis), and the villous wall became irritated. The rest of the feed was seen accumulating in the intestine, which suggests that feed containing high FFM (40%) is not properly absorbed, but rather causes intestinal damage.

## Discussion

Protease and lipase enzyme activity was higher at the end of the experiment than at the beginning. This is in contrast to amylase, for which activity was higher at the beginning ^19^. The activity of protease, lipase, and amylase enzymes of silver pompano can be detected during the larva stage, and tends to increase with age ^20^. Protease activity in batik grouper larvae ( *Epinephelus microdon*) was shown to increase with age ^21^. Enzyme activity tends to increase with fish age because with age the digestive organs develop and enter the definitive phase; therefore, the consumption of exogenous feed is increased, which is a source of energy to trigger enzyme activity. In addition, high activity is related to the role of the pancreas in secreting enzymes. When an enzyme is secreted in small amounts, the activity will be low. However, when it is secreted in large amounts, the activity will increase.

Amylase activity was seen to be lower at the end than at the beginning of the observation ^19^. The activity increased up to the 24th day, then decreased to the 30th day of observation ^14^. The decrease in enzyme activity is influenced by the length of its reaction with the substrate. A longer reaction causes the enzyme to lose some of its activity. The reduced amylase activity in fish is also influenced by low concentrations in the digestive tract or concentrations that have exceeded the optimal limit. Furthermore, this decrease might be due to the feeding habits of carnivorous fish such as silver pompano which is lower amylase activity than protease and lipase enzymes. Hence, even though the fish consumes a high amount of the substrate, as long as the amylase concentration is low, the activity will not increase.

The results also showed that the protease enzyme activity of fish fed with FFM (P1, P2, P3 and P4) was higher than those without FFM (P0), and was significantly different (P <0.05) between the treatments. The highest protease enzyme activity was found in 30% FFM feed and was significantly different from other treatments (p<0.05). Jayadi ^20^ stated that the increase in protease activity is caused by the exogenous feed consumed, which stimulates the increase in digestive enzyme activity. The production of these enzymes is strongly influenced by the amount of protein in the feed. In addition, Lundstedt *et al.* ^22^ stated that protease activity is strongly influenced by the amount of active protease, the feed, and the quality of the diet.

The feed that contained no FFM in this study was able to stimulate an increase in lipase enzyme activity. However, the more FFM (40%) in the feed, the higher the lipase activity was found to be, and activity was significantly different between treatments (p<0.05). This is supported by Duc *et al.* ^23^, who stated that some *Bacillus* species produce extracellular enzymes such as proteases, lipases, amylase, and cellulases that facilitate feed digestion. In addition, several factors that influence enzyme activity according to Yamin and Palinggi ^14^ are the nature of the substrate, the type of enzyme, and the environmental conditions in the digestive tract.

Amylase activity was higher with increasing FFM in the feed, and was significantly different (p<0.05) between treatments. Chor *et al.* ^24^ stated that activity is influenced by feed composition, carbohydrate content, and fish feeding habits. Chor *et al.* ^24^ also stated that the lipase and protease activity of omnivorous fish was higher than amylase. Furthermore, omnivorous species of fish have amylase levels and an amylase-protease ratio that is higher than those of carnivorous types.

Feed without FFM (P0) had the lowest digestibility compared to those with FFM (P1, P2, P3, and P4). Feed containing 20% FFM (P2) had the highest digestibility but was not significantly different (p>0.05) from feed containing 30% FFM (P3). Mazotto *et al.* ^4^ and Brandelli *et al.* ^6^ stated that *B. subtilis* produces protease, lipase, and amylase that are useful in improving the quality of proteins and breaking down complex nutrients into simple absorbable molecules, thereby increasing digestibility in feed without FFM ^25^. Digestibility values of artificial feed depends on the level of fish reception and the available enzymes. Therefore, the addition of exogenous enzyme has the potential to optimize feed digestibility. In addition, when the protein, lipid, and carbohydrate content correspond to the enzyme activity, digestibility will increase.

Feed that contained FFM had a higher protein digestibility than those without it. This is supported by Zerdani *et al.* ^26^, who stated that processing feather meal with *B. licheniformis* increased the digestibility of the protein by 54.20%. The results of this study showed that feed and protein digestibility are best obtained in fish fed with 20% FFM (P2) ^27^. The digestibility of proteins is determined by exogenous and endogenous factors. The exogenous factors are the interactions of proteins with polyphenols, carbohydrates, lipids, and protease inhibitors. The endogenous factors are related to the characterization of protein structures such as tertiary, quaternary, and structures that can be damaged by heat. When compared with the results of Adelina *et al.* ^8^, in which feed containing 10% FFM fermented by *B. subtilis* in white snapper produced 48.75% protein digestibility, the value of this study was higher (67.24%).

Feed without FFM (P0) had lipid digestibility that was not significantly different (p>0.05) from that of feed containing FFM (P1, P2, P3 and P4). Marzuqi and Anjusary ^28^ stated that high or low digestibility is influenced by lipase enzyme activity. Therefore, the more the lipid in the feed, the higher the activity of lipase. Furthermore, digestibility is influenced by several factors including protein and carbohydrate components, the process of making the feed, the particle size, type and size of fish and the amount of feed consumed ^25^.

Feed without FFM (P0) had the lowest carbohydrate digestibility compared to those containing FFM (P1, P2, P3, and P4) and was significantly different (p<0.05), whereas those containing FFM (P1, P2, P3 and P4) had carbohydrate digestibility that was not significantly different between treatments (p>0.05). This correlated with higher amylase activity in fish fed with FFM compared to those without FFM. Marzuqi and Anjusary ^28^ stated that fish do not have adequate carbohydrate digestive enzymes in their digestive tract, hence the digestibility value of this nutrient is generally low. However, FFM fermentation using *B. subtilis* in this study was able to produce higher amylase activity and increase the digestibility of carbohydrates.

Fish fed with no FFM (P0) showed a normal intestine on histological examination, and can therefore properly digest feed ^29^. The intestine has an important role in food digestion, especially in nutrient absorption. Fujaya ^30^ stated that nutrients are not directly absorbed, but are first broken down into their simpler components in the form of amino acids, fatty acids, and glucose. Specifically for proteins, the degradation process occurs in the stomach by pepsin and in the intestine by trypsin ^30^. Meanwhile, fish intestine after treatment P1 (10% FFM) was seen to produce goblet cells. These cells produce mucus, which has a role in facilitating absorption and transportation of feed molecules through membranes, as well as providing protection against micro-organisms in the intestine ^31^. The test feed containing 10% FFM was seen to be absorbed by the intestine of silver pompano ^9^. Feather protein degraded by the *B. subtilis* enzyme could be digested and effectively absorbed by the intestines of broiler chickens, and did not damage their intestinal function. However, the intestine of P1 fish showed congestion. Kalaiyarasi *et al.* ^32^ stated that this is an event of vessel dilation due to increased blood volume in the circulatory system.

The intestine of fish fed with a diet containing 20% FFM (P2) produced more goblet cells, which means they effectively absorb nutrients ^18^. Fish digestive activity requires a lot of enzyme secretion; therefore, the intestine stimulates goblet cells to produce more mucus to protect the outer lining against damage and irritation ^33^. Probiotic bacteria regulate the microbial environment in fish intestines and block pathogenic micro-organisms by releasing amylase, protease, cellulase, and lipase, which help to hydrolyze nutrients. They also facilitate the breakdown of carbohydrates, proteins, and lipid into smaller molecules.

The use of 30% FFM in feed (P3) resulted in congestion and hemorrhage of the fish intestinal tissue. This is characterized by erythrocyte leakage in the mucus membrane. The hemorrhage that occurs in the intestine can be caused by several agents such as foreign materials or objects that enter into the digestive tract, causing damage to the wall ^34^.

In fish fed with 40% FFM (P4), the intestine had more severe damage (necrosis) and the villi wall was irritated. Furthermore, the rest of the feed piled up in the intestine, which showed that 40% FFM is quite high and is not properly absorbed, causing damage. The necrosis that occurs is characterized by the appearance of damaged tissue ^18^. Necrosis is death of a cell or tissue after an advanced stage of degeneration. This can be caused by trauma or an interruption in blood supply in certain areas ^34^. The necrotic tissues have several characteristics including an abnormal pale color, brittleness, and poor consistency. Hence, erosion or irritation of intestinal villi causes the loss of some epithelium in the lining of mucosa, causing it to become thinner. This will in turn cause disruption of nutrients absorption, leading to malnutrition and even death ^34^.

## Conclusion

The use of FFM in feed resulted in increased activity of digestive enzymes (protease, lipase) in silver pompano. The more FFM there was in the feed, the higher the activities of the enzymes were. The digestibility of nutrients (proteins, lipids, and carbohydrates) in feed that contained FFM was better than in feed without FFM. While 20% FFM in feed was efficiently digested and absorbed, 40% FFM could not be digested or absorbed, but rather caused intestinal irritation and necrosis.

## Data availability

Figshare: Raw data of Cr2O3 and proximate composition of diets. https://doi.org/10.6084/m9.figshare.13109486.v1 ^35^.

Figshare: Raw data of enzyme activity. https://doi.org/10.6084/m9.figshare.13109495.v1 ^36^.

Figshare: Raw data of feed and nutrient digestibility. https://doi.org/10.6084/m9.figshare.13109501.v1 ^37^.

Figshare: Intestinal histology. https://doi.org/10.6084/m9.figshare.13109498.v1 ^38^.

Data are available under the terms of the Creative Commons Attribution 4.0 International license (CC-BY 4.0).
