# Supplementary material for: Use of chicken feather meal fermented with Bacillus subtilis in diets to increase the digestive enzymes activity and nutrient digestibility of silver pompano Trachinotus blochii (Lacepede, 1801)
Source: F1000Res. 2021 Mar 25;10:25. Originally published 2021 Jan 15. [Version 2] doi: 10.12688/f1000research.26834.2 (PMC8030120; doi:10.12688/f1000research.26834.2)
Supplement: Peer review history

# Peer review: Reviewer response for version 2

**DOI:** 10.5256/f1000research.55545.r82250

**Published:** 2021-04-06

**Authors:** Rudy Agung Nugroho

**Affiliations:** 1 Animal Physiology, Development and Molecular Laboratory, Department of Biology, Faculty of Mathematic and Natural Science, Mulawarman University, Samarinda, Indonesia

I am glad to read that the manuscript has now improved significantly from the previous version. The corrections which have been made by the authors are satisfactory. I can now recommend this paper for indexing. Best of luck for the future research.

Is the work clearly and accurately presented and does it cite the current literature?

Yes

If applicable, is the statistical analysis and its interpretation appropriate?

Yes

Are all the source data underlying the results available to ensure full reproducibility?

Partly

Is the study design appropriate and is the work technically sound?

Yes

Are the conclusions drawn adequately supported by the results?

Yes

Are sufficient details of methods and analysis provided to allow replication by others?

Partly

Reviewer Expertise:

Animal/Fish Physiology, Fish nutrition, Biochemistry.

I confirm that I have read this submission and believe that I have an appropriate level of expertise to confirm that it is of an acceptable scientific standard.

# Peer review: Reviewer response for version 2

**DOI:** 10.5256/f1000research.55545.r82251

**Published:** 2021-04-01

**Authors:** Christopher Marlowe A. Caipang

**Affiliations:** 1 Department of Biology, College of Liberal Arts, Sciences, and Education, University of San Agustin, Iloilo City, Philippines

I have  read the revisions made by the authors and they have addressed the issues that I raised in the previous version of the submission. They have also cited some of the limitations that they encountered in the conduct of the experiment. However, they should take into consideration these limitations and should minimize these in their future works.

Is the work clearly and accurately presented and does it cite the current literature?

Partly

If applicable, is the statistical analysis and its interpretation appropriate?

Yes

Are all the source data underlying the results available to ensure full reproducibility?

Partly

Is the study design appropriate and is the work technically sound?

Yes

Are the conclusions drawn adequately supported by the results?

Yes

Are sufficient details of methods and analysis provided to allow replication by others?

Partly

Reviewer Expertise:

Fish health management, immunology

I confirm that I have read this submission and believe that I have an appropriate level of expertise to confirm that it is of an acceptable scientific standard.

# Peer review: Reviewer response for version 1

**DOI:** 10.5256/f1000research.29631.r79933

**Published:** 2021-03-05

**Authors:** Rudy Agung Nugroho

**Affiliations:** 1 Animal Physiology, Development and Molecular Laboratory, Department of Biology, Faculty of Mathematic and Natural Science, Mulawarman University, Samarinda, Indonesia

Review report for the work entitle: Use of chicken feather meal fermented with *Bacillus subtilis* in diets to increase the digestive enzymes activity and nutrient digestibility of silver pompano *Trachinotus blochii* (Lacepede, 1801).

 This work is informative, has a great result, and benefit for the knowledge of fisheries fields. However, there are some points should be addressed to the author(s) and need to be clarified.

**Title**

**Abstract**

*Background*: please add scientific name of silver pompano for the first appear in this section.

*Methods*: the term P1, P2, P3, P4 can be deleted, it is unused in this abstract.

*Conclusion* **, **please be more specific, it is stated that the addition of Chicken feather meal fermented with *B. subtilis* could increase the activity of digestive enzyme, but the result found that the amylase activity of silver pompano was reduced.

**Introduction**

 Acceptable.

**Materials and Methods**

1. It is stated that “ The sterile outcome was placed in five Petri dishes (2 g each), adding 10 ml of pure *B. subtilis*, and incubating at 50°C, pH 8 for 72 hours 12. The FFM was then ready to be used as fish feed ingredient.” Does the FFM that was resulted from five Petri dishes was enough for the whole study?
2. Please mention the density of fish in each plastic tank, and how much water in each plastic tank.
3. Please be specific on how the protease, amylase and lipase activity were determined, there should be any of equations.
4. In analysis of intestine histology: it is mention that the observations were then compared to determine the differences between the treatments. Please state what variable that was compared among the treatments (Goblet, histopathology?).

**Results**

 Adequate.

**Discussion**

 Please discuss why amylase activity was lower at the end of the study in relation with FFM. This in contrast with the statement that feed with FFM had the higher digestibility, though was not significantly different among treatments.

**Conclusion**

 Sufficient.

**References**

 Sufficient.

Is the work clearly and accurately presented and does it cite the current literature?

Yes

If applicable, is the statistical analysis and its interpretation appropriate?

Yes

Are all the source data underlying the results available to ensure full reproducibility?

Partly

Is the study design appropriate and is the work technically sound?

Yes

Are the conclusions drawn adequately supported by the results?

Yes

Are sufficient details of methods and analysis provided to allow replication by others?

Partly

Reviewer Expertise:

Animal/Fish Physiology, Fish nutrition, Biochemistry.

I confirm that I have read this submission and believe that I have an appropriate level of expertise to confirm that it is of an acceptable scientific standard, however I have significant reservations, as outlined above.

# Response

**Published:** 2021-03-17

**Authors:** Indra Suharman

**Affiliations:** University of Riau, Indonesia

1. Comment :

 Please add scientific name of silver pompano for the first appear in this section.

 Respond :

 We have added this information in the background of abstract.

 2. Comment :

 The term P1, P2, P3, P4 can be deleted, it is unused in this abstract.

 Respond :

 We have deleted this information in the method of abstract.

 3. Comment :

 Please be more specific, it is stated that the addition of Chicken feather meal fermented with *B. subtilis* could increase the activity of digestive enzyme, but the result found that the amylase activity of silver pompano was reduced.

 Respond :

 We have explained more specific this statement in the conclusion of abstract.

 4. Comment :

 It is stated that “ The sterile outcome was placed in five Petri dishes (2 g each), adding 10 ml of pure *B. subtilis*, and incubating at 50°C, pH 8 for 72 hours 12. The FFM was then ready to be used as fish feed ingredient.” Does the FFM that was resulted from five Petri dishes was enough for the whole study?

 Respond :

 We only used the FFM that was resulted from five petri dishes for the proximate composition analysis after fermentation.

 5. Comment :

 Please mention the density of fish in each plastic tank, and how much water in each plastic tank.

 Respond :

 We have added this information in the material and method section.

 6. Comment :

 Please be specific on how the protease, amylase and lipase activity were determined, there should be any of equations.

 Respond :

 We have added this information in the material and method section.

 The activity of protease, amylase and lipase enzymes were determined by following methods that were previously used by Bergmeyer et al. (1983), Borlongan (1990) and Bernfield (1955) respectively which the equations were involved in these methods.

 7. Comment :

 In analysis of intestine histology: it is mention that the observations were then compared to determine the differences between the treatments. Please state what variable that was compared among the treatments (Goblet, histopathology?).

 Respond :

 We have mentioned this information in the material and method section.

 8. Comment :

 Please discuss why amylase activity was lower at the end of the study in relation with FFM. This in contrast with the statement that feed with FFM had the higher digestibility, though was not significantly different among treatments.

 Respond :

 We have developed the discussionas suggested by the reviewer in the discussion section.

# Peer review: Reviewer response for version 1

**DOI:** 10.5256/f1000research.29631.r77534

**Published:** 2021-02-08

**Authors:** Christopher Marlowe A. Caipang

**Affiliations:** 1 Department of Biology, College of Liberal Arts, Sciences, and Education, University of San Agustin, Iloilo City, Philippines

This manuscript describes the use of chicken feather meal fermented with *Bacillus subtilis* to increase digestibility and digestive enzyme activity in pompano. Results showed that FFM enhanced digestive protease and lipase but not amylase. Supplementation with 20% fermented FFM resulted in higher digestibility of nutrients.

 The results presented are straightforward. However, there are certain issues in the methodology that I think the authors need to address:

1. Please provide data that the fermentation of feather meal with *Bacilus* resulted in the partial breakdown down the composition.
2. For the nutrient digestibility studies, how did the authors prevent leaching of the nutrients from feces during collection?
3. The initial levels of amylase were high. Theoretically, the levels of the digestive enzymes will be more or less similar with the P0 levels. In the case of amylase, there is a wide discrepancy between P0 and the initial. Please explain this phenomenon.
4. Kindly explain why no analyses were done within the 60 day of feeding. An analysis for 2-3 sampling points within the feeding period will give us a better picture of the trends of the enzyme activity.
5. Please explain why very few fish were used for the experiment.
6. Please provide a better resolution of the histological slides that will clearly show changes in some of the morphological/cellular structures to indicate the effect of the FFM.

I hope that these issues will be addressed by the authors when they revise their manuscript.

Is the work clearly and accurately presented and does it cite the current literature?

Partly

If applicable, is the statistical analysis and its interpretation appropriate?

Yes

Are all the source data underlying the results available to ensure full reproducibility?

Partly

Is the study design appropriate and is the work technically sound?

Yes

Are the conclusions drawn adequately supported by the results?

Yes

Are sufficient details of methods and analysis provided to allow replication by others?

Partly

Reviewer Expertise:

Fish health management, immunology

I confirm that I have read this submission and believe that I have an appropriate level of expertise to confirm that it is of an acceptable scientific standard, however I have significant reservations, as outlined above.

# Response

**Published:** 2021-03-18

**Authors:** Indra Suharman

**Affiliations:** University of Riau, Indonesia

1. Comment:

 Please provide data that the fermentation of feather meal with *Bacilus* resulted in the partial breakdown down the composition.

 Respond:

 We have published the data of fermentation of feather meal in IOP Conference Series : Earth and Environmental Science (2019).

 2. Comment:

 For the nutrient digestibility studies, how did the authors prevent leaching of the nutrients from feces during collection?

 Respond:

 To prevent leaching of the nutrients from feces during collection, we directly collected the feces that are released by the fish by siphoning (siphoning method).

 3. Comment:

 The initial levels of amylase were high. Theoretically, the levels of the digestive enzymes will be more or less similar with the P0 levels. In the case of amylase, there is a wide discrepancy between P0 and the initial. Please explain this phenomenon.

 Respond:

 This phenomenon might be affected by feeding habits and biochemical composition of diet. Other studies indicated that the amylase activity largely depends on the feeding habits of fish. We have added more discussion in the discussion section.

 4. Comment:

 Kindly explain why no analyses were done within the 60 day of feeding. An analysis for 2-3 sampling points within the feeding period will give us a better picture of the trends of the enzyme activity.

 Respond:

 Because we only want to know the enzyme activity at the beginning and the end of the experiment.

 5. Comment:

 Please explain why very few fish were used for the experiment.

 Respond:

 We have used a total of 150 fish that were distributed into 15 identical 20-L plastic jars at a density of 10 fish per jar. We have made a corrrection in the material and method section.

 6. Comment:

 Please provide a better resolution of the histological slides that will clearly show changes in some of the morphological/cellular structures to indicate the effect of the FFM.

 Respond:

 We only have this resolution of the histological slides.
